# The efficacy of ultrasound-guided radiofrequency ablation for the treatment of papillary thyroid carcinoma

**DOI:** 10.12669/pjms.41.10.12649

**Published:** 2025-10

**Authors:** Bin Wang, Fuqiang Zhang, Fan Sun

**Affiliations:** 1 Bin Wang Department of Interventional Medicine, Xinchang County People’s Hospital, Shaoxing, Zhejiang Province 312500, P.R. China; 2 Fuqiang Zhang Department of Nail and Breast Surgery, Xinchang County People’s Hospital, Shaoxing, Zhejiang Province 312500, P.R. China; 3 Fan Sun Ultrasound Department, Yuhuan Second People’s Hospital, Taizhou City, Zhejiang Province 317605, China

**Keywords:** Conventional surgical, Papillary thyroid carcinoma, Radiofrequency ablation, Ultrasound-guided

## Abstract

**Objective:**

This study compared the clinical value of ultrasound (US)-guided radiofrequency ablation (RFA) and conventional surgical treatment for papillary thyroid carcinoma (PTMC).

**Methodology:**

This retrospective cohort study included clinical data of PTMC patients who underwent routine surgical procedures or US-guided RFA treatment at Xinchang County People’s Hospital between April 2023 to July 2024. Perioperative status (surgical duration, intraoperative blood loss, length of hospital stay), changes in the levels of thyroid-related hormones and inflammatory factors and incidence of complications were compared.

**Results:**

A total of 125 patients met the criteria for this study. Among them, 60 patients underwent US-guided RFA (RFA group) and 65 underwent routine surgical procedures (surgical group). RFA was associated with better perioperative status than conventional surgery (P<0.05). After the surgery, the levels of thyroid-stimulating hormone (TSH) in both groups increased compared to preoperative levels and were significantly higher in the surgical group compared to the RFA group. The levels of free thyroxine (FT4) and free triiodothyronine (FT3) decreased compared to preoperative levels and were considerably lower in the surgical group than in the RFA group (P<0.05). Postoperative levels of C-reactive protein (CRP), interleukin-6 (IL-6) and tumor necrosis factor - α (TNF - α) in both groups demonstrated a significant increase compared to preoperative level and were markedly higher in the surgical group compared to the RFA group (P<0.05). RFA was associated with a significantly lower incidence of complications compared to conventional surgery (5.00% vs 16.92%, respectively) (P<0.05).

**Conclusions:**

US-guided RFA for treating PTMC is more effective than conventional surgical procedures and is associated with better perioperative status and levels of thyroid-related hormones, lower inflammatory response and lower incidence of complications.

## INTRODUCTION

Papillary thyroid microcarcinoma (PTMC) refers to a papillary thyroid carcinoma with a diameter of ≤ 10 mm and no distant lymph node metastasis or thyroid invasion.^1^,^2^ PTMC usually has no specific clinical manifestations and patients are diagnosed during physical examinations.^2^ PTMC accounts for about 85% of the total number of thyroid cancer cases and is associated with a good prognosis.^1^-^3^ Early and safe treatment is, therefore, significant in ensuring disease efficacy and prognosis.^4^,^5^

While thyroidectomy is considered the standard treatment for PTMC, the procedure can easily cause damage to adjacent structures, such as the recurrent laryngeal nerve and parathyroid gland, leading to related complications.^6^,^7^ In recent years, with the widespread application of thyroid ultrasound (US), US-guided fine needle aspiration has become increasingly popular.^3^,^4^ US-guided RFA has the advantages of minimal damage, short treatment time, precise efficacy, minimal impact on surrounding structures and patient aesthetics, fast recovery after ablation and no need for lifelong medication.^8^ Currently, US-guided RFA is mainly used to treat benign thyroid nodules.^8^,^9^ While recent studies have shown that US-guided RFA also has good therapeutic effects on PTMC patients, there is currently no consensus on its clinical value.^9^

Among thermal ablation techniques for PTMC, radiofrequency ablation (RFA), laser ablation (LA), and microwave ablation (MWA) are the most commonly investigated. While LA and MWA can achieve effective tumor necrosis, RFA offers real-time ultrasound guidance with high procedural precision, a favorable safety profile, and broader clinical adoption.^10^ RFA is supported by large-scale studies and long-term follow-up demonstrating durable local control in low-risk PTMC, whereas the evidence base for LA and MWA is comparatively more limited. In addition, RFA devices are widely available in routine practice and procedural protocols are relatively standardized.^6^,^10^ Given these considerations—and because surgery remains the standard of care—we designed this study to directly compare perioperative outcomes, thyroid function preservation, systemic inflammatory response, and complication rates between RFA and conventional surgery in patients with low-risk PTMC.

This study compared the efficacy and complications of US-guided RFA and conventional surgical intervention in treating PTMC. Our results may assist in defining a treatment method that minimizes trauma while ensuring treatment effectiveness.

## METHODOLOGY

This retrospective case-control study included clinical records of PTMC patients who underwent routine surgical procedures or US-guided RFA treatment at Xinchang County People’s Hospital from April 2023 to July 2024.

### Ethical Approval:

The ethics committee of Xinchang County People’s Hospital approved this study with the number 2024-K-013-01, Date: September 3^rd^, 2024.

### Inclusion criteria:

- Meet the diagnostic criteria for PTMC.^1^
- Diagnosed through US-guided fine needle aspiration biopsy.
- There is no obvious abnormality in the contralateral thyroid gland.
- No lymph node metastasis or cervical lymph node enlargement.
- Complete clinical data.

### Exclusion criteria:

- Patients with abnormal vocal cord function on the contralateral side of the tumor.
- Patients with large calcifications in tumors.
- Patients with coagulation dysfunction.
- Patients with a history of radiation and chemotherapy treatment.
- Ultrasound examination or cell puncture indicates capsule invasion.

### Conventional surgical treatment:

The patient was placed in the supine position, with shoulders elevated and head tilted backward and general anesthesia was administered. An incision was made about 4-6 cm above the collarbone. The thyroid gland was exposed, the affected thyroid isthmus and lobes were removed and the cervical lymph nodes were cleared. After the conventional placement of the drainage tube, the incision was closed.

### US-guided RFA therapy:

The procedure used SIEMENS OXANA2 probe, ultrasonic instrument and 18G RFA needle. The patient was assisted in the supine position, with a soft pillow behind the neck and instructed to stretch the neck as much as possible. Subcutaneous injection of lidocaine (1%) was injected, followed by local anesthesia along the needle path to the anterior capsule of the thyroid gland. The decision whether to use isolation fluid was based on the location of the lesion. Targeted ablation was performed and the ablation range was increased appropriately. The duration of single-point ablation was about 30-45 seconds and the RF ablation output power was set to 20-30 W. Care was taken to determine that the gasification range during surgery was about 5 mm larger than the edge of the lesion.

### Data collection:

- Basic characteristics, including age, gender, lesion diameter, nodule location and calcification status.
- Perioperative situation, including surgical duration, intraoperative blood loss and length of hospital stay.
- Serum thyroid-related hormone levels, including thyroid stimulating hormone (TSH), free thyroxine (fT4) and free triiodothyronine (fT3), were detected by chemiluminescence using CL-6000i fully automatic chemiluminescence immunoassay analyzer (manufacturer: Mindray, Shenzhen, China).
- Levels of serum inflammatory cytokine indicators, including CRP, IL-6 and TNF - α were measured by enzyme-linked immunosorbent assay (kit manufacturer: BOSTER; Wuhan, China).
- The occurrence of complications.

### Statistical Analysis:

The data were analyzed using SPSS version 26.0 (IBM Corp, Armonk, NY, USA). According to the Shapiro-Wilk test, the distribution normality was evaluated; normal distribution data were represented by mean ± standard deviation (SD) and an independent sample t-test was used for inter-group comparison. Non-normally distributed data were represented by a median and interquartile range and the Whitney U test was used for inter-group comparison. The count data were represented by the number of cases using the chi-square test. P<0.05 was statistically significant.

## RESULTS

The study included clinical records of 125 patients (60 males and 65 females), with an average age of 47.42±7.90. Of them, 60 patients underwent US-guided RFA treatment and 65 received conventional surgical treatment, with no statistically significant difference in the basic characteristics between the two groups (P>0.05) Table-I.

**Table-I:** Comparison of Basic Characteristics between Two Groups.

Item	RFA group (n=60)	Surgery group (n=65)	t/χ^2^/Z	P
Age (year), mean±SD	48.57±8.12	46.37±7.60	1.563	0.121
***Sex, n (%)***				
Male	26 (43.33)	34 (52.31)	1.007	0.316
Female	34 (56.67)	31 (47.69)		
Disease diameter (mm), M(P25/P75)	5 (5-6.5)	6 (5-8)	-1.740	0.082
***Nodule location, n (%)***				
Right lobe of thyroid gland	26 (43.33)	34 (52.31)	1.398	0.497
Left lobe of thyroid gland	24 (40.00)	24 (36.92)		
Isthmus of thyroid gland	10 (16.67)	7 (10.77)		
Calcification (yes), n (%)	41 (68.33)	49 (75.38)	0.769	0.380

**Fig.1:**
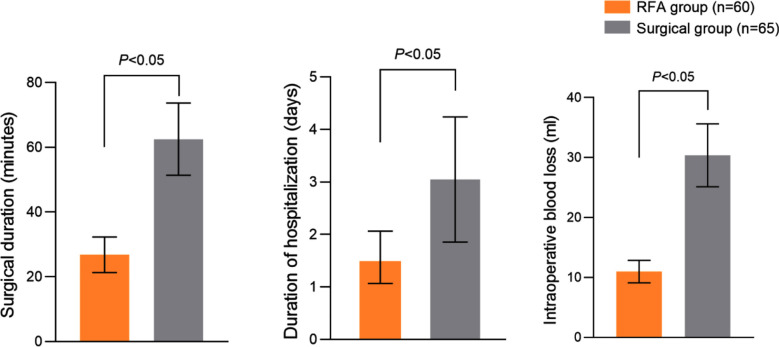
Comparison of surgical duration, hospitalization duration and intraoperative blood loss between two groups.

The RFA group had shorter surgical duration and hospitalization time compared to the surgical group and the intraoperative blood loss was significantly lower than that of the surgical group (P<0.05) Fig.1.

**Fig.2:**
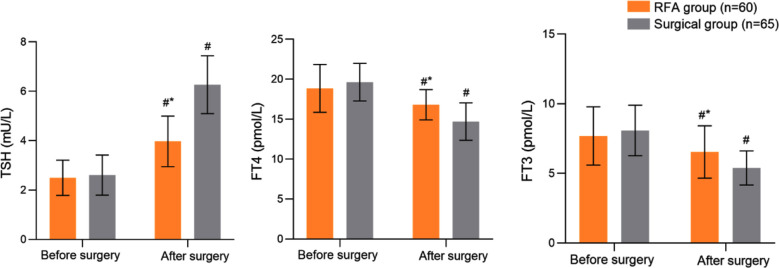
Comparison of thyroid hormone levels between two groups; Compared with preoperative results in the same group, ^#^*P*<0.05; Compared with the surgical group, *P<0.05; TSH: thyroid stimulating hormone; FT4: free thyroxine; FT3: free triiodothyronine.

Before the surgery, serum TSH, FT4 and FT3 levels were comparable in the two groups (P>0.05). After the surgery, TSH levels in both groups increased significantly compared to preoperative levels and were considerably higher in patients who underwent conventional surgery than those who underwent RFA. In contrast, postoperative fT4 and fT3 levels significantly decreased and were lower in the surgical group compared to the RFA group (P<0.05) Fig.2.

**Fig.3:**
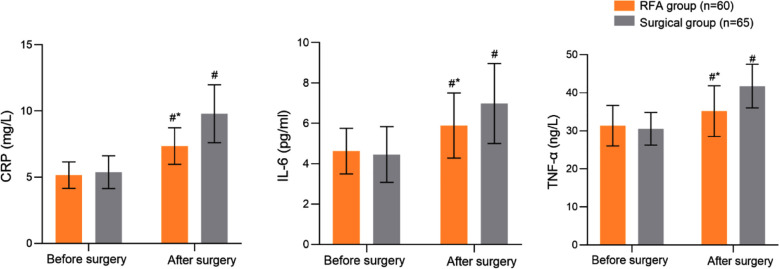
Comparison of inflammatory cytokine levels between two groups; Compared with preoperative results in the same group, ^#^*P*<0.05; Compared with the surgical group, *P<0.05; CRP: C-reactive protein; IL-6: interleukin-6; TNF - α: tumor necrosis factor - α.

No significant difference in the preoperative levels of serum CRP, IL-6 and TNF - α were detected in the two groups (P>0.05). Postoperative serum CRP, IL-6 and TNF - α in both groups were significantly increased and were markedly higher in the surgical group patients compared to the RFA group (P<0.05). Fig.3. The incidence of complications in the RFA group (5.00%) was lower than that in the surgery group (16.92%) (P<0.05) Table-II. In the surgery group, complications included transient hoarseness (n=3, 4.62%), pain requiring additional analgesics (n=3, 4.62%), parathyroid injury with postoperative hypocalcemia requiring calcium supplementation (n=3, 4.62%), and wound infection treated with antibiotics (n=1, 1.54%). In the RFA group, complications were limited to transient hoarseness (n=1, 1.67%), mild pain (n=1, 1.67%), and suspected parathyroid irritation (n=1, 1.67%), all of which resolved with conservative management.

**Table-II:** Comparison of incidence of complications between two groups.

Group	n	Hoarseness	Heating	Pain	Parathyroid injury	Infect	Total incidence rate
RFA group	60	1 (1.67)	0 (0.00)	1 (1.67)	1 (1.67)	0 (0.00)	3 (5.00)
Surgery group	65	3 (4.62)	1 (1.54)	3 (4.62)	3 (4.62)	1 (1.54)	11 (16.92)
*χ^2^*							4.460
*P*							0.035

## DISCUSSION

This study compared the efficacy of conventional surgical procedures and US-guided RFA in treating PTMC patients. It showed that RFA is associated with significantly shorter surgical duration and hospital stay and lesser intraoperative blood loss than the conventional surgery.

Our results are consistent with the research conclusions of Zheng L et al.^11^ and Seo YK et al.^12^ Compared with surgical procedures, US-guided RFA directly and precisely applies high-frequency current to the lesion tissue, achieving local high-temperature coagulation necrosis, while avoiding direct cutting and suturing of blood vessels. Such an approach significantly reduces the risk of intraoperative bleeding and facilitates early postoperative recovery.^12^,^13^

Our study also showed that postoperative TSH levels increased, while FT4 and FT3 levels decreased in both groups compared to preoperative levels. Our results indicate that both methods impact the thyroid function of patients. However, the TSH levels in the RFA group were lower, while the FT4 and FT3 levels were higher than those in the surgery group. Our results are consistent with the findings of Ren Y et al.,^14^ which showed that compared to conventional surgical procedures, US-guided RFA therapy has a smaller impact on thyroid function in patients with PTMC. The observed postoperative increase in TSH and decrease in FT4 and FT3 in both groups suggest a reduction in functional thyroid tissue. If sustained, these changes may predispose patients to subclinical or overt hypothyroidism, potentially requiring lifelong levothyroxine supplementation, and could influence metabolism, cardiovascular health, mood, and quality of life.^14^,^15^ While our short-term results indicate better preservation of thyroid function after RFA, the long-term clinical consequences remain unclear. Future prospective studies with extended follow-up are needed to evaluate the association between postoperative thyroid hormone alterations, patient-reported outcomes, and overall prognosis. Cesareo et al.^16^ also showed that RFA is a minimally invasive treatment method that allows for maintaining the relative stability of thyroid hormones and reduces the occurrence of postoperative complications such as hypothyroidism.

This study also showed that while the levels of inflammatory markers such as serum CRP, IL-6 and TNF - α increased after the surgery in both groups of patients, they were significantly lower in patients who underwent US-RFA compared to the surgery group. Our results indicate that the inflammatory response caused by US-guided RFA is relatively mild. We may speculate that the minimally invasive nature of US-guided RFA can reduce the area of surgical trauma and alleviate the degree of inflammation and stress response in the body. Previous studies have also demonstrated that such reduced stress response is beneficial for reducing postoperative pain and accelerates postoperative functional recovery.^17^,^18^

The incidence of complications is an essential indicator for evaluating treatment safety. This study demonstrated that the incidence of complications in the RFA group was significantly lower than in the surgical group. Our results further demonstrate the safety advantages of US-guided RFA. The lower incidence of complications not only reduces patient stress but also lowers the demand and cost of subsequent treatment. Our results are consistent with the conclusions of multiple clinical studies.^19^,^20^ Cao XJ et al.^19^ showed that RFA therapy can reduce the incidence of complications and improve the safety of treatment, providing strong evidence for the safety of US-guided RFA in clinical applications. Han ZY et al.^20^ also showed that the incidence of complications in RFA therapy for PTMC was significantly lower than that of the conventional surgical procedures and most of the complications were mild and easy to manage, which is consistent with the conclusion of our study. Traditional surgical procedures require extensive dissection, which may lead to complications such as recurrent laryngeal nerve damage, hypoparathyroidism and postoperative bleeding. In contrast, the non-invasive nature of RFA prevents many of the risks and reduces the occurrence of complications through precise US-guided treatment.^20^ Some surgical complications, such as recurrent laryngeal nerve injury and parathyroid dysfunction, may be related to the extent of tissue dissection and individual patient factors (e.g., anatomic variability or comorbidities).^21^ The less invasive nature of RFA may therefore account for its lower complication rate observed in our cohort.

The clinical advantages observed with RFA—shorter hospitalization, milder systemic inflammatory response, and fewer complications—are likely to translate into meaningful improvements in patients’ quality of life (QoL), including faster return to normal activities, less postoperative discomfort, and reduced cosmetic or endocrine concerns.^22^ Although our database did not include standardized patient-reported outcome measures (e.g., ThyPRO, SF-36), published studies on RFA for low-risk PTMC have reported favorable patient satisfaction and quicker functional recovery compared with surgery. Future prospective studies should incorporate validated QoL instruments and economic endpoints to quantitatively assess these benefits.

This study provides new evidence from a real-world cohort directly comparing RFA and surgery in low-risk PTMC, incorporating perioperative metrics, systemic inflammatory markers, postoperative thyroid function, and complication profiles. Our results support the role of RFA as an organ-preserving alternative in appropriately selected cases, with the potential to reduce perioperative morbidity and preserve endocrine function. Strengths of this study include the direct head-to-head design in a homogeneous patient population, the inclusion of multiple clinically relevant outcomes, and the use of standardized operative and ablative protocols performed by experienced operators. Future research should focus on multi-center prospective trials with extended follow-up to assess recurrence, sustained thyroid function, and patient-reported quality of life, thereby defining the optimal role of RFA in PTMC management.

### Limitations:

This single-center retrospective study, with a relatively small sample size, is inherently susceptible to selection bias and unmeasured confounding, limiting causal inference. To mitigate this risk, we consecutively enrolled patients meeting strict predefined eligibility criteria, ensured comparable baseline demographic and tumor characteristics between groups, and used standardized operative or ablative protocols performed by experienced operators. Nevertheless, variability in operator expertise, intraoperative decision-making, and patient-specific factors (e.g., anatomical variations, comorbidities, rehabilitation compliance) could not be fully eliminated. Comorbidities such as diabetes and hypertension were not systematically recorded and thus could not be adjusted for, representing a potential source of residual confounding. The follow-up period was relatively short, precluding direct assessment of long-term outcomes such as recurrence and sustained thyroid function after RFA. Existing long-term studies, however, report durable local control and recurrence rates below 5% at 5–10 years in appropriately selected low-risk PTMC patients. Future multi-center prospective trials with extended follow-up, randomization or propensity score matching, and comprehensive comorbidity assessment are warranted to confirm these findings and better define long-term endocrine outcomes. Complications were recorded without standardized severity grading or uniform long-term follow-up, and thus we reported only overall rates and main types rather than detailed prognostic implications. Prospective studies should incorporate validated classification systems (e.g., Clavien–Dindo) and prolonged follow-up to assess their clinical impact. Finally, the applicability of RFA is limited to patients with solitary PTMC ≤10 mm, without capsule invasion, extrathyroidal extension, cervical lymph node metastasis, or significant contralateral lobe abnormalities. It is less suitable for multifocal disease, lesions abutting critical structures where safe thermal margins cannot be achieved, extensive macrocalcification, larger tumors, or features suggestive of advanced disease. The safety and oncologic efficacy of RFA in these complex scenarios require further prospective evaluation.

## CONCLUSION

The results of this study provide important references for the treatment of PTMC. We show that the US-guided RFA can shorten surgical duration and reduce hospitalization time and intraoperative blood loss. Moreover, RFA has shown significant advantages in protecting thyroid function, reducing inflammatory reactions and lowering the incidence of complications.

### Authors’ contributions:

**BW:** Study design, literature search and manuscript writing.

**BW and FZ:** Data collection, data analysis and interpretation. Critical analysis

**BW and FS:** Manuscript revision and validation and is responsible for the integrity of the study.

All authors have read and approved the final manuscript.
